# A Swarm Optimization Genetic Algorithm Based on Quantum-Behaved Particle Swarm Optimization

**DOI:** 10.1155/2017/2782679

**Published:** 2017-05-25

**Authors:** Tao Sun, Ming-hai Xu

**Affiliations:** ^1^College of Pipeline and Civil Engineering, China University of Petroleum, Qingdao 266580, China; ^2^Shengli College, China University of Petroleum, Dongying, Shandong 257000, China

## Abstract

Quantum-behaved particle swarm optimization (QPSO) algorithm is a variant of the traditional particle swarm optimization (PSO). The QPSO that was originally developed for continuous search spaces outperforms the traditional PSO in search ability. This paper analyzes the main factors that impact the search ability of QPSO and converts the particle movement formula to the mutation condition by introducing the rejection region, thus proposing a new binary algorithm, named swarm optimization genetic algorithm (SOGA), because it is more like genetic algorithm (GA) than PSO in form. SOGA has crossover and mutation operator as GA but does not need to set the crossover and mutation probability, so it has fewer parameters to control. The proposed algorithm was tested with several nonlinear high-dimension functions in the binary search space, and the results were compared with those from BPSO, BQPSO, and GA. The experimental results show that SOGA is distinctly superior to the other three algorithms in terms of solution accuracy and convergence.

## 1. Introduction

Particle swarm optimization (PSO) algorithm is a population-based optimization method, which was originally introduced by Eberhart and Kennedy in 1995 [1]. In PSO, the position of a particle is represented by a vector in search space, and the movement of the particle is determined by an assigned vector called the velocity vector. Each particle updates the velocity based on its current velocity, the best previous position of the particle, and the global best position of the population. PSO is extensively used for the optimization problems because it has simple structures and is easy to implement. However, it has some disadvantages, such that it easily falls into local optima when solving the complex and high-dimension problems [2, 3]. Hence a number of variant algorithms have been proposed to overcome the disadvantages of PSO [4, 5].

The particle swarm algorithm based on the probability convergence is one of the variant algorithms. This kind of particle swarm algorithm allows the particles to move according to probability instead of using velocity-displacement particle movement way. The Bare Bones PSO (BBPSO) family is a typical class of probabilistic PSO algorithms [6–8]. The Gaussian distribution was used in the original version of BBPSO, which was proposed by Kennedy [6]. then several new BBPSO variants used other distributions which seem to generate better results [7–9].

Inspired by the quantum theory and the trajectory analysis of PSO [10], Sun et al. proposed a new probabilistic algorithm, quantum-behaved particle swarm optimization (QPSO) algorithm [11]. In QPSO, each particle has a target point, which is defined as a linear combination of the best previous position of the particle and the global best position. The particle appears around the target point following a double exponential distribution. The QPSO algorithm essentially belongs to the BBPSO family, and its update equation uses an adaptive strategy and has fewer parameters to be adjusted [12–14]. The QPSO has been shown to perform well in finding the optimal solutions for continuous optimization problems and successfully applied to a wide range of areas such as multiobjective optimization [15, 16], clustering [17–19], neural network training [20–22], image processing [23, 24], engineering design [25], and dynamic optimization [26].

PSO and QPSO have been effective tools for solving global optimization problems, but they were originally developed for continuous search spaces. Kennedy and Eberhart introduced a binary version of PSO for discrete problems named binary PSO (BPSO) [27], where the trajectories are defined as changes in the probability that each particle changes its state to 1. Binary PSO has simple structure and is easy to implement; hence, it is extensively employed in the optimization problems [28–30]. But it also suffers from some disadvantages when solving the complex and high-dimension problems [28]. Sun et al. proposed binary QPSO (BQPSO), in which the target point is obtained by using the crossover operator at the best previous position of the particle and the global best position. Experiment results show that BQPSO can find better solution generally than BPSO [31].

In recent years, BQPSO has been used successfully in many fields [32–34]. However, although BQPSO broadens the application fields of QPSO, it did not show the same advantage as in the continuous space. QPSO algorithm should have better performance in solving the problems based on discrete space. This paper analyzes the main factors that impact the search ability of QPSO and converts the particle movement formula to the mutation condition by the introduction of rejection region. It then designed a new binary coding QPSO, which has crossover and mutation operator and is like genetic algorithm (GA) in form; that is, the proposed algorithm is a new genetic algorithm but incorporates the core idea of QPSO. So it was named swarm optimization genetic algorithm (SOGA).

Compared with the GA, the SOGA has no selection operator, and each individual participates in evolution based on the information of the population and its own information. At the same time, the mutation probability of the SOGA is not fixed. In the early stage of the algorithm, the probability of mutation is large and the population can keep the diversity, with the iteration of the algorithm, the mutation probability tends to zero, and the algorithm can finally converge.

The rest of this paper is organized as follows.  Section 2 is a brief introduction of PSO and binary PSO; Section 3 summarizes QPSO and binary QPSO; Section 4 introduces the mutation condition of binary coding converted from the particle movement formula in QPSO; Section 5 proposes the new binary QPSO algorithm, SOGA, and then discusses the difference between this algorithm and QPSO, GA; Section 6 presents the experiment results from the benchmark functions; finally, the paper is concluded in Section 7.

## 2. Particle Swarm Optimization

Particle swarm optimization (PSO) algorithm is a population-based optimization technique used in continuous spaces. It can be mathematically described as follows.

Assume the size of the population is *n* and the dimension of the search space is *q*; then the *i*th particle of the swarm can be represented by a position vector *X*_*i*_ = (*x*_*i*1_, *x*_*i*2_,…, *x*_*iq*_); the velocity of a particle *i* is denoted by vector *V*_*i*_ = (*v*_*i*1_, *v*_*i*2_,…, *v*_*iq*_); vector *P*_*i*_ = (*p*_*i*1_, *p*_*i*2_,…, *p*_*iq*_) is the best previous position of particle *i*, called personal best position, and *P*_*g*_ = (*p*_*g*1_, *p*_*g*2_,…, *p*_*gq*_) is the best position of the population, called global best position.

The velocity of particle *i* is calculated accordingly:

(1) $$\begin{array}{c} v_{ij}^{k+1}=\omega v_{ij}^{k}+c_{1}r_{1}\left(\;p_{ij}^{k}-x_{ij}^{k}\;\right)+c_{2}r_{2}\left(\;p_{gj}^{k}-x_{ij}^{k}\;\right), \end{array}$$

where *i* = 1,2,…, *n*, *j* = 1,2,…, *q*,  *n* is population size, *k* is the number of iterations, *ω* is inertia weight, *c*_1_ and *c*_2_ are acceleration coefficients, and *r*_1_ and *r*_1_ are random numbers in the interval [0,1].

Then the next position is updated as follows:

(2) $$\begin{array}{c} x_{ij}^{k+1}=x_{ij}^{k}+v_{ij}^{k+1}. \end{array}$$

The PSO algorithm is applied to solve optimization problems in the real search space, but many optimization problems are set in discrete space. Kennedy and Eberhart proposed a discrete binary version of PSO, named binary PSO (BPSO), where the particle position has two possible values, “0” or “1.” The velocity formula in BPSO remains unchanged, and the particle position is updated as follows:

(3) $$\begin{array}{c} x_{ij}^{k+1}=\left\{\begin{array}{cc} 1 & S\left(v_{ij}^{k+1}\right)>rand\\ 0 & otherwise, \end{array}\right. \end{array}$$

where rand is a random number in the interval [0,1] and the function *S*(*v*) is a Sigmoid function as

(4) $$\begin{array}{c} S\left(\;v\;\right)=\frac{1}{\left(1+e^{-v}\right)}. \end{array}$$

## 3. Quantum-Behaved Particle Swarm Optimization

Inspired by trajectory analyses of PSO in [10], Sun et al. proposed a novel variant of PSO, named quantum-behaved particle swarm optimization (QPSO), which outperforms the traditional PSO in search ability.

QPSO sets a target point for each particle; denote *G*_*i*_ = (*g*_*i*1_, *g*_*i*2_,…, *g*_*iq*_) as the target point for particle *i*, of which the coordinates are

(5) $$\begin{array}{c} g_{ij}=\phi_{ij}p_{ij}+\left(\;1-\phi_{ij}\;\right)p_{gj}, \end{array}$$

where *ϕ*_*ij*_ is a random number in the interval [0,1]. the trajectory analysis in [10] shows that *G*_*i*_ is the local attractor of particle *i*; that is, in PSO, particle *i* converges to it.

The position of particle *i* is updated as follows:

(6) xijk+1=gijk±Lijk2ln⁡1u,Lijk=2αcj−xijk,

where *u* is a random number in the interval [0,1] and *C* = [*c*_1_, *c*_1_,…, *c*_*q*_] is known as the mean best position that is defined by the average of the personal best position of all particles, accordingly,

(7) $$\begin{array}{c} c_{j}=\frac{1}{n}\overset{n}{\underset{t=1}{\sum }}p_{tj},\;j=1,2,\ldots ,q, \end{array}$$

Parameter *α* is called Contraction-Expansion Coefficient, which can be tuned to control the convergence speed of the algorithms.

Because the iterations of QPSO are different from those of PSO, the methodology of BPSO cannot be applied to QPSO. Sun et al. introduced the crossover operator of GA into QPSO and proposed binary QPSO (BQPSO). In BQPSO, *X*_*i*_ = (*x*_*i*1_, *x*_*i*2_,…, *x*_*iq*_) still represents the position of particle *i*, but it is necessary to emphasize that *X*_*i*_ is a binary string rather than a vector, and *x*_*ij*_ is the *j*th substring of *X*_*i*_, not the *j*th bit in the binary string. Assume the length of each substring is *l*; then the length of *X*_*i*_ is *lq*.

The target point *G*_*i*_ for particle *i* is generated through crossover operator; that is, BQPSO exerts crossover operation on the personal best position *P*_*i*_ and the global best position *P*_*g*_ to generate two offspring binary strings, and *G*_*i*_ is randomly selected from them.

Define

(8) $$\begin{array}{c} p_{m}=\alpha *d_{H}\left(\;c_{j},x_{ij}^{k}\;\right)*\ln\left(\;\frac{1}{u}\;\right),\;u\sim U\left[0,1\right], \end{array}$$

where *k* is the number of iterations and *d*_*H*_(*c*_*j*_, *x*_*ij*_^*k*^) is the Hamming distance between *c*_*j*_ and *x*_*ij*_^*k*^. Compared with the two bit strings, the Hamming distance is the count of bit difference in the two strings. *c*_*j*_ is the *j*th substring of the mean best position, and the *d*th bit of *c*_*j*_ is determined by the states of the *d*th bit of all particles' personal best positions. If more particles take on 1 at the *d*th bit, the *d*th bit of *c*_*j*_ is 1; otherwise the bit will be 0.

For each bit of *g*_*ij*_, when *p*_*m*_ > rand execute operations as follows: if the state of the bit is 1, then set its state to 0; else set its state to 0.

## 4. A Mutation Condition Using in Binary Space

The reason why the QPSO algorithm has better global search capability than the traditional PSO algorithm is that it changes the velocity-displacement model of the traditional PSO algorithm; in QPSO, the movement of particle to its target point has no determined trajectory; it can appear at any position in the whole feasible search space with a certain distribution, which is the double exponential distribution [13, 14]. Such a position can be far from the target point and may be superior to the current global best position of the population. This should also be reflected in the construction of binary QPSO algorithm.

The probability density function of particle *i* in QPSO is

(9) $$\begin{array}{c} f\left(\;X_{i}\;\right)=\frac{1}{L_{i}}e^{-2\left|X_{i}-G_{i}\right|/L_{i}},\;\left(L_{i}>0\right),\;\;i=1,2,\ldots ,n. \end{array}$$

Set *λ*_*i*_ = 2/*L*_*i*_, and *y*_*i*_ = *X*_*i*_ − *G*_*i*_; then (9) can be rewritten as

(10) $$\begin{array}{c} f\left(\;y_{i}\;\right)=\frac{\lambda_{i}}{2}e^{-\lambda_{i}\left|y_{i}\right|},\;\left(\lambda_{i}>0\right). \end{array}$$

**Figure 1:**
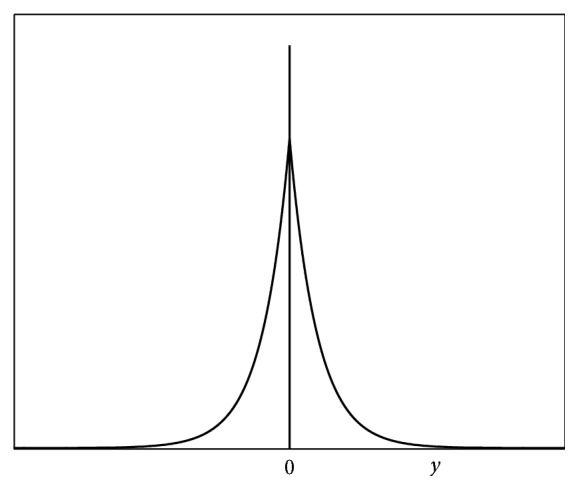
Probability density function of double exponential distribution.

That is, *y*_*i*_ obeys the double exponential distribution, of which the mean and variance are *E*(*y*_*i*_) = 0 and *D*(*y*_*i*_) = 2/*λ*_*i*_^2^. The graph of probability density function (10) is Figure 1. Since the domain of *y*_*i*_ is (−*∞*, +*∞*), particle can appear in any position of the search space, but the probability that a particle appears in a position far away from its target point is small. When *λ*_*i*_ → +*∞*, the variance *D*(*y*_*i*_) = 2/*λ*_*i*_^2^ → 0 which means that *X*_*i*_ converge to *G*_*i*_ with probability 1.

**Figure 2:**
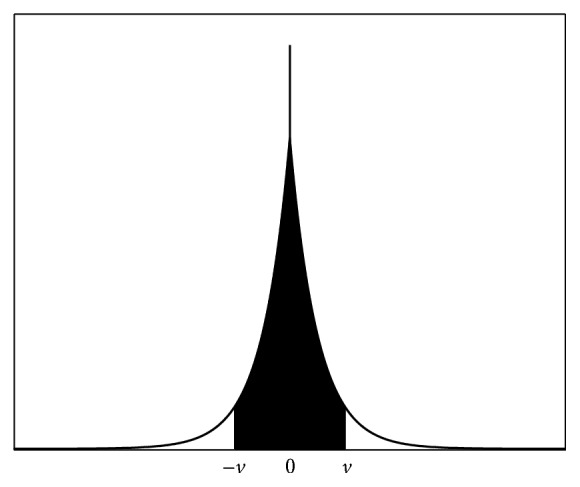
Refused domain of probability density function.

When the position of a particle uses binary encoding, it is hard to describe the relative position of two points using the measure of two binary strings. Similar to set a rejection region, we set a threshold value *v*  (*v* > 0). When the value of *y*_*i*_ falls into the rejection region, as shown in Figure 2, set *y*_*i*_ = 0; that is, *X*_*i*_ = *G*_*i*_, else *X*_*i*_ = mutation(*G*_*i*_). mutation(*G*_*i*_) means mutation operation on *G*_*i*_.

For any *u*, which is a random number in the interval [0,1], the condition that *y*_*i*_ does not fall into the rejection region is

(11) $$\begin{array}{c} P\left(\;\left|\;y_{i}\;\right|>v\;\right)>u. \end{array}$$

The left side of Condition (11) can be written as

(12) Pyi>v∫−∞−vλi2eλitdt+∫v+∞λi2e−λitdt=2∫v+∞λi2e−λitdt=e−λiv.

Thus Condition (11) means *e*^−*λ*_*i*_*v*^ > *u*, accordingly:

(13) $$\begin{array}{c} \lambda_{i}v<\ln\left(\;\frac{1}{u}\;\right). \end{array}$$

In order to ensure that the algorithm can converge, set

(14) $$\begin{array}{c} \lambda_{i}=\frac{1}{d_{H}\left(C,X_{i}\right)}, \end{array}$$

where *C* is the mean best position of the population.

Then Condition (13) is

(15) $$\begin{array}{c} \frac{v}{d\left(C,X_{i}\right)}<\ln\left(\;\frac{1}{u}\;\right), \end{array}$$

where *d*(·) is used to measure the difference of two binary strings. Hamming distance can be used here.

Assume *y* = ln⁡(1/*u*); then

(16) $$\begin{array}{c} u=exp\left(\;-y\;\right),\;y>0. \end{array}$$

**Figure 3:**
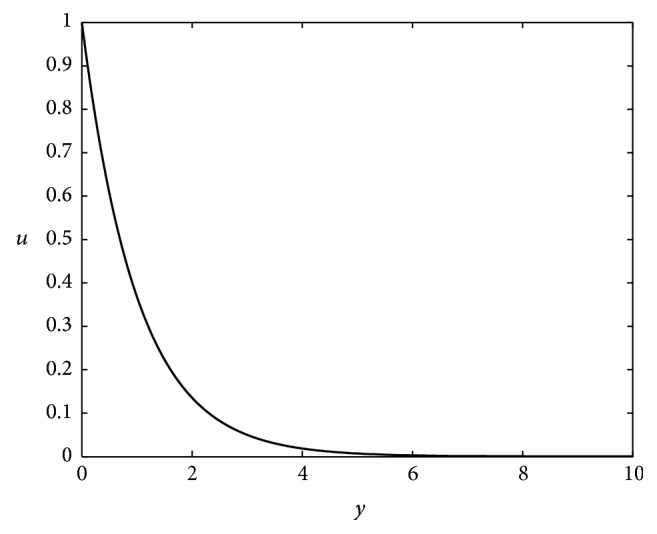
Figure of equation (16).

For (16), when the value of *y* is small, the function has fast rates of change as shown in Figure 3, so Condition (15) suffers from the effect of the initial value of *d*(*C*, *X*_*i*_). So Condition (15) can be changed into its equivalent form:

(17) $$\begin{array}{c} \sigma d\left(\;C,X_{i}\;\right)>\ln\left(\;\frac{1}{u}\;\right), \end{array}$$

where parameter *σ* is a constant that is greater than zero.

## 5. Swarm Optimization Genetic Algorithm

Based on the mutation condition (17), mutation operator is introduced into BQPSO. *X*_*i*_ still represents the position of particle *i*, *P*_*i*_ is the personal best position of particle *i*, *P*_*g*_ is the global best position, and *C* is the mean best position which is defined the same as in BQPSO.

Different from BQPSO, crossover or mutation operation process is applied to the whole binary string, instead of bits. Because the procedure of the algorithm is similar to GA, it is named as swarm optimization genetic algorithm (SOGA). The process can be described as follows.

1. Initialize a population of particles *X*_*i*_ in binary space;
2. Set personal best position *P*_*i*_ = *X*_*i*_, and compute *C*;
3. Evaluate the fitness of particles *f*(*X*_*i*_) and determine the global best position *P*_*g*_;
4. while terminate condition is not reached do
5. for each particle *i* do
6. Exert crossover operation on *P*_*i*_ and *P*_*g*_ to generate two offspring binary strings,
7. *G*_*i*_ is randomly selected from them.
8. if condition (17) is true,
9. Exert mutation operation on *G*_*i*_;
10. end if
11. Set *X*_*i*_ = *G*_*i*_;
12. Compute the fitness of particles *f*(*X*_*i*_), and update *P*_*i*_,
13. end for *i*
14. Update *P*_*g*_ and the mean best position *C*;
15. end while

Compared to the GA with the same crossover and mutation operator, SOGA has the following characteristics:

(1) SOGA does not have selection operator and crossover probability and its crossover operator is exerted directly on *P*_*i*_ and *P*_*g*_. Therefore, the form of the fitness function *f*(*X*_*i*_) has no effect on the algorithm, and the target function of the maximization problem can be set as the fitness function.
(2) Condition (17) can be turned into
  (18) $$\begin{array}{c} u>exp\left(\;-\sigma d\left(\;C,X_{i}\;\right)\;\right). \end{array}$$
Since *σd*(*C*, *X*_*i*_) ≥ 0, the range of exp(−*σd*(*C*, *X*_*i*_)) is (0,1), and *u* is a random number in the interval [0,1]; thus Condition (18) is equivalent to an adaptive mutation probability:
  (19) $$\begin{array}{c} p_{m}=1-exp\left(\;-\sigma d\left(\;C,X_{i}\;\right)\;\right), \end{array}$$
where *σ* is a constant that is greater than zero and *d*(*C*, *X*_*i*_) decreases with the increase of iteration times. Therefore, *p*_*m*_ is shrunk, which causes the algorithm to converge.
(3) *σ* is the only parameter of SOGA, which can be tuned to control the convergence speed of the algorithms as Contraction-Expansion Coefficient *α* in BQPSO.

**Figure 4:**
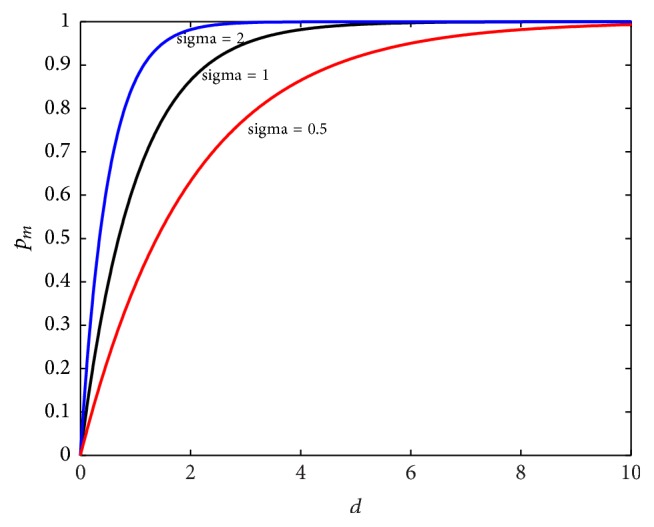
Figures of mutation probability.

When the value of *σ* is 0.5, 1, and 2, the curves of mutation probability *p*_*m*_ changing with *d*(·) are shown in Figure 4. The figure demonstrates that the smaller the value of *σ*, the faster the convergence speed of the algorithm. It also can be seen that the global searching ability of the algorithm is reduced when *σ* is too small. So set *σ* = 1 in SOGA.

## 6. Experimental Results

The proposed SOGA is compared with BPSO, BQPSO, and GA. They are tested on the following 10 benchmark problems to be minimized [28, 35]:

(1) Sphere Function

(20) $$\begin{array}{c} F_{1}\left(\;X\;\right)=\overset{n}{\underset{i=1}{\sum }}x_{i}^{2},\;\left|x_{i}\right|\leq 100. \end{array}$$

(2) Schwefel's Problem 2.22

(21) $$\begin{array}{c} F_{2}\left(\;X\;\right)=\overset{n}{\underset{i=1}{\sum }}\left|\;x_{i}\;\right|+\overset{n}{\underset{i=1}{\prod }}\left|\;x_{i}\;\right|,\;\left|x_{i}\right|\leq 10. \end{array}$$

(3) Schwefel's Problem 1.2

(22) $$\begin{array}{c} F_{3}\left(\;X\;\right)=\overset{n}{\underset{i=1}{\sum }}{\left(\;\sum_{j=1}^{i}x_{j}\;\right)}^{2},\;\left|x_{i}\right|\leq 100. \end{array}$$

(4) Step Function

(23) $$\begin{array}{c} F_{4}\left(\;X\;\right)=\overset{n}{\underset{i=1}{\sum }}{\left(\;\left\lfloor \;x_{i}+0.5\;\right\rfloor \;\right)}^{2},\;\left|x_{i}\right|\leq 100. \end{array}$$

(5) Schwefel's Problem 2.21

(24) $$\begin{array}{c} F_{5}\left(\;X\;\right)=\underset{i}{max}\left\{\;\left|\;x_{i}\;\right|\;\right\},\;i=1,2,\ldots ,n,\;\;\left|x_{i}\right|\leq 100. \end{array}$$

(6) 2^*n*^ Minima Function

(25) $$\begin{array}{c} F_{6}\left(\;X\;\right)=\frac{1}{n}\overset{n}{\underset{i=1}{\sum }}\left(\;x_{i}^{4}-16x_{i}^{2}+5x_{i}\;\right),\;\left|x_{i}\right|\leq 5. \end{array}$$

(7) Schwefel's Problem 1.2

(26) $$\begin{array}{c} F_{7}\left(\;X\;\right)=\overset{n}{\underset{i=1}{\sum }}{-x}_{i}\sin\left(\;\sqrt{\left|x_{i}\right|}\;\right),\;\left|x_{i}\right|\leq 500. \end{array}$$

(8) Ackley Function

(27) F8X=−20exp⁡−0.2∑i=1nxi2n−exp∑i=1ncos2πxin+20+e,xi≤32.

(9) Generalized Penalized Function

(28) F9X=πn10sin⁡πy1+∑i=1n−1yi−121+10 sin2⁡πyi+1+yn−12+∑i=1nuxi,10,100,4,yi=1+xi+14,  uxi,a,k,m=kxi−amxi>a,0−a≤xi≤a,k−xi−amxi<−a.  xi≤50

(10) Griewank Function

(29) F10X=14000∑i=1nxi2−∏i=1ncosxii+1,xi≤600.

In these functions, *F*_1_ ~ *F*_5_ are unimodal and *F*_6_ ~ *F*_10_ are multimodal. Their optimum values are all zeros except *F*_6_ and *F*_7_. The minimum values of *F*_6_ and *F*_7_ are −78.3323 and −418.9829*∗n*, respectively, where *n* is the dimension of a function.

In the experiments, the dimension of each function is 8, and the binary code length of each continuous variable is 15, so the length of particle is 120 for each function. The size of population is 50 and the total number of iterations is set to 500. The parameters of algorithms are listed in Table 1, where *p*_*c*_ is crossover probability and *p*_*m*_ is mutation probability in GA.

Four algorithms ran independently 30 times on the benchmark functions, and the best target function value was recorded at each run. To compare the four algorithms, 30 data sets were analyzed using the following statistic parameters: the mean, the standard deviation (STD), the best, the worst, and the median; these results are reported in Tables 2 and 3.

Moreover, the statistical test is conducted in order to determine whether the average best results are different with a statistical significance. The confidence level is fixed at 0.95, and the tests return *p* value which are shown in Tables 4 and 5. We use the SAS for statistical testing; in the SAS system, if the *p* value is less than 0.0001, the system displays <0.0001. The value of *h* in Tables 4 and 5 shows the result of pairwise comparison; *h* = 1 indicates the previous comparison algorithm is significantly better than the latter; *h* = 0 represents no significant difference between the two compared algorithms; *h* = −1 indicates the previous comparison algorithm is significantly worse than the latter.

**Figure 5:**
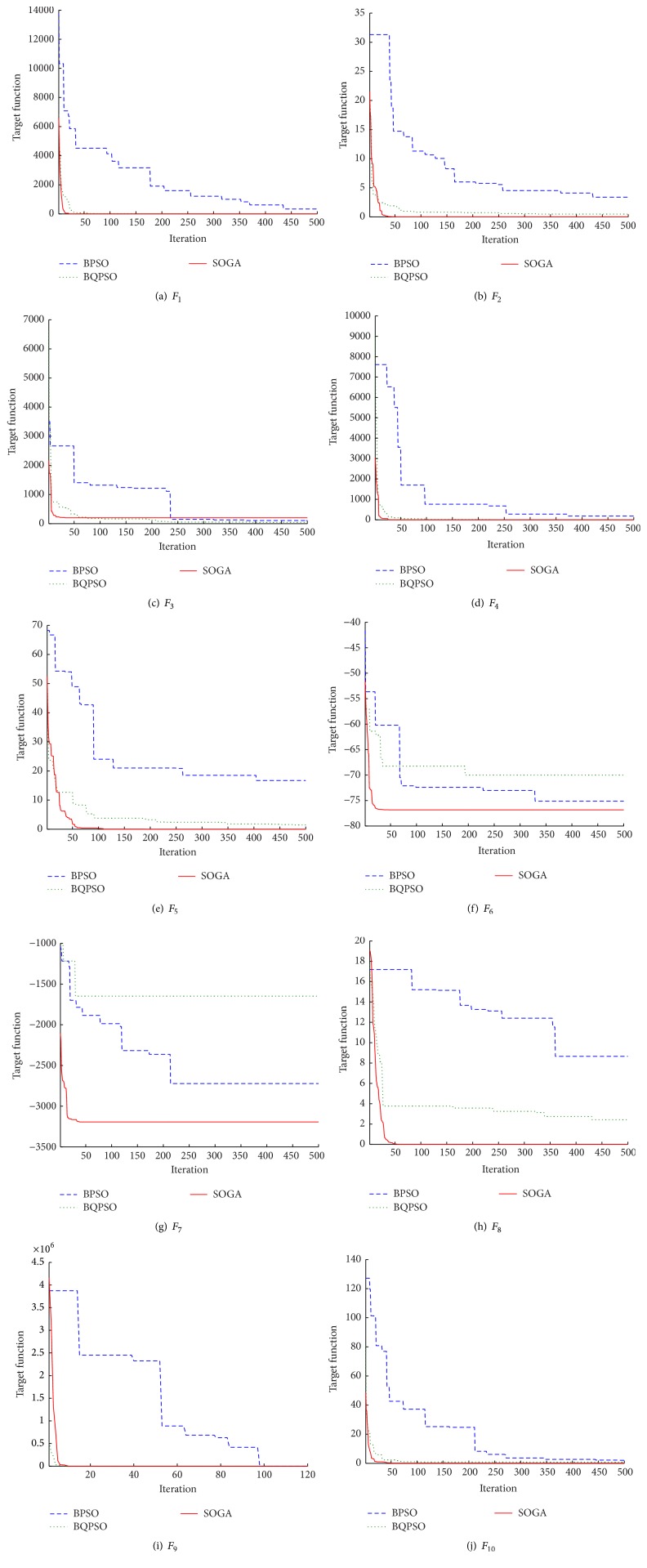
Figures of the convergence processes of BPSO, BQPSO, and SOGA.

The results of SOGA compared with BPSO and BQPSO are listed in Tables 2 and 4. The results show that SOGA surpasses BPSO and BQPSO in minimizing the ten benchmark functions except *F*_3_.  Figure 5 illustrates the convergence process of the best target function value of population in one running. As shown in Figure 5, the SOGA converges faster than BPSO and BQPSO.

Since SOGA has almost the same form as GA, the same crossover and mutation operator, single-point crossover and single-point mutation, are used in both algorithms. In GA, the elitist strategy is applied to improve the convergence and optimization results. It should be noted that the GA can not converge after 500 iterations for most of the functions; for better comparison, the number of iterations of the GA is set to 2000 in Table 3 to ensure that the algorithm is fully convergent.

For high-dimension functions, assume *X*_*i*_ = (*x*_*i*1_, *x*_*i*2_,…, *x*_*iq*_) is the binary string of the particle (or individual) *i*, where *q* is the number of dimensions and *x*_*ij*_ is the *j*th substring of *X*_*i*_. It is easy for GA or SOGA to exert crossover and mutation operation on each substring *x*_*ij*_ in turn, instead of on the whole *X*_*i*_. For instance, in SOGA, Condition (17) can be written as

(30) $$\begin{array}{c} \sigma d\left(\;c_{j},x_{ij}\;\right)>\ln\left(\;\frac{1}{u}\;\right), \end{array}$$

for each substring *x*_*ij*_ of *X*_*i*_, where *c*_*j*_ are the *j*th substring of *C*. Then the process of SOGA when the crossover and mutation operation act on substring can be described as follows: the same operation can also be used in GA.

1. Initialize a population of particles *X*_*i*_ in binary space;
2. Set personal best position *P*_*i*_ = *X*_*i*_, and compute *C*;
3. Evaluate the fitness of particles *f*(*X*_*i*_) and determine the global best position *P*_*g*_;
4. while terminate condition is not reached do
5. for each particle *i* do
6. for each substring of particle *j* do
7. Exert crossover operation on *P*_*ij*_ and *P*_*gj*_ to generate two offspring binary strings,
8. *G*_*ij*_ is randomly selected from them.
9. if condition (30) is true,
10. Exert mutation operation on *G*_*ij*_;
11. end if
12. Set *X*_*ij*_ = *G*_*ij*_;
13. end for *j*
14. Compute the fitness of particles *f*(*X*_*i*_), and update *P*_*i*_,
15. end for *i*
16. Update *P*_*g*_ and the mean best position *C*;
17. end while

**Figure 6:**
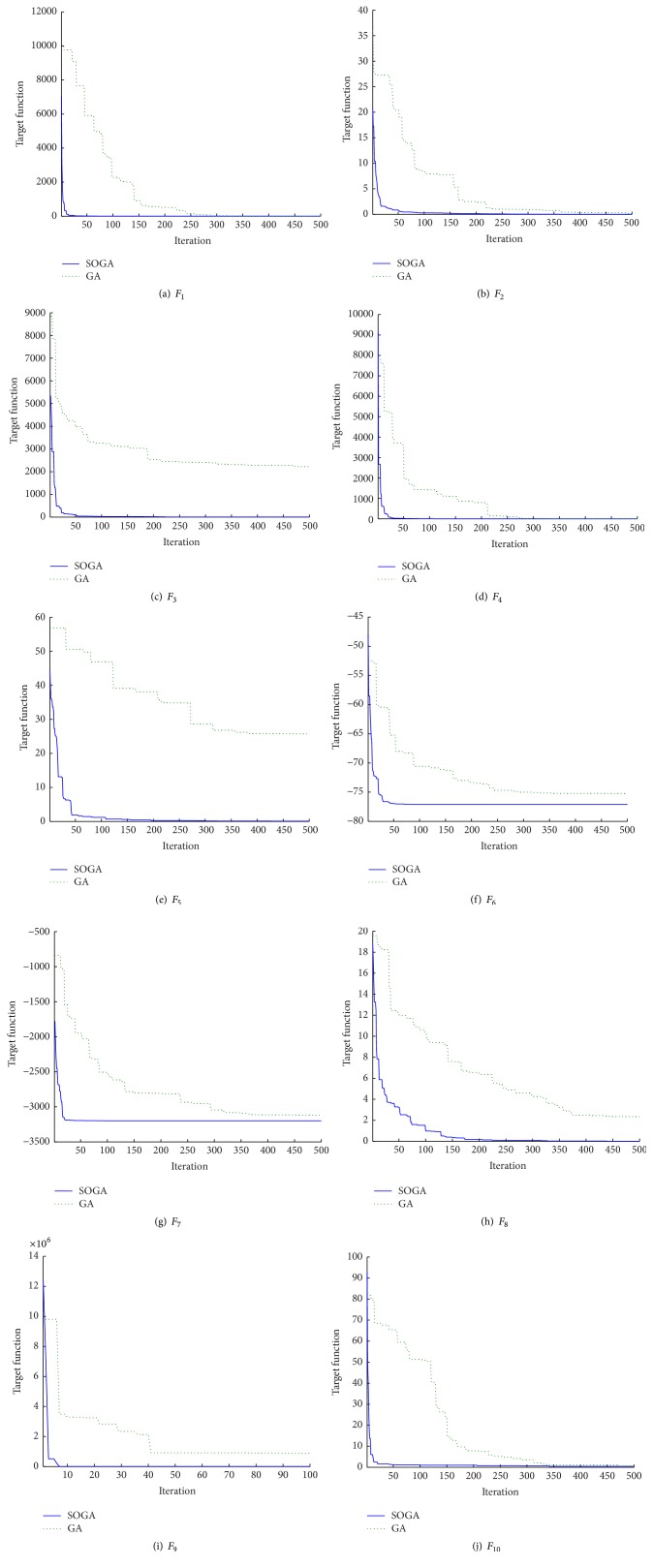
Figures of the convergence processes of SOGA and GA when the crossover and mutation operation act on substring.

The convergence processes of SOGA and GA, when crossover and mutation operation act on substrings of particles (or individual), are shown in Figure 6; the results of SOGA and GA are listed in Tables 3, 4, and 5. The experimental results show that SOGA is obviously superior to the GA on the solution accuracy and the convergence. For high-dimension functions, it is effective to improve the convergence speed and optimization ability, exerting crossover and mutation operation on substrings; as shown in Tables 3 and 4, it significantly improves the convergence rate of GA. But its influence is not significant for SOGA; according to Table 5, it has better performance in minimizing functions *F*_3_, *F*_7_, *F*_8_, and *F*_9_, especially for function *F*_3_.

## 7. Conclusions

In this study, SOGA, a binary swarm intelligence algorithm, which is based on QPSO and binary QPSO, is introduced. It converts the movement formula of QPSO to mutation conditions, thus introducing the mutation operator of GA. SOGA has the similar form to GA but does not need to set the crossover and mutation probability, so it has fewer parameters to control. SOGA integrates strongpoint of GA and PSO. The experimental results show that SOGA is distinctly superior to BPSO, BQPSO, and GA in terms of solution accuracy and convergence. Furthermore, since SOGA has the same crossover and mutation operator as GA, many improvements on the GA can be applied to it; therefore, this algorithm has better applications and research prospects.

## Figures and Tables

**Table 1:** Parameters of algorithms applied in the experiments.

Algorithm	Parameter settings
SOGA	*σ* = 1
BPSO	*ω* = 0.7, *c*_1_ = *c*_2_ = 2, *V*_max_ = 6
BQPSO	*α* = 1.1~1.4
GA	*p*_*c*_ = 0.90, *p*_*m*_ = 0.10~0.15

**Table 2:** Minimization results for BPSO, BQPSO, and SOGA.

Function	Algorithm	The best	Mean	SD	The worst	Median
*F*_1_	BPSO	65.1674	278.1128	160.0441	702.2221	244.4259
	BQPSO	1.4096	3.3647	1.4272	6.5327	3.1107
	SOGA	7.4510*e* − 05	1.6641*e* − 04	4.8945*e* − 04	0.0028	7.4510*e* − 05

*F*_2_	BPSO	1.5918	3.0268	0.9300	5.3401	2.9463
	BQPSO	0.2155	0.4080	0.1226	0.7025	0.4025
	SOGA	0.0024	0.0026	0.0005	0.0049	0.0024

*F*_3_	BPSO	121.8395	1171.3480	911.2165	3261.0044	860.7055
	BQPSO	8.2112	30.8752	25.8621	120.6034	21.4084
	SOGA	263.9170	1919.6076	1078.9650	3782.1623	2175.0068

*F*_4_	BPSO	56	312.7667	248.9619	1154	250.5
	BQPSO	1	4.8667	2.4174	11	5
	SOGA	0	0.1000	0.4026	2	0

*F*_5_	BPSO	9.0243	16.3089	5.5677	27.5491	14.3406
	BQPSO	0.9430	1.8437	0.5141	3.3296	1.8342
	SOGA	0.0031	0.2228	0.6241	3.1281	0.0275

*F*_6_	BPSO	−77.5179	−74.5146	1.8144	−70.6644	−74.5941
	BQPSO	−72.7114	−69.3385	1.7327	−66.4601	−68.9703
	SOGA	−77.7357	−76.2789	0.6355	−75.1676	−76.2048

*F*_7_	BPSO	−3136.9403	−2724.3377	194.8185	−2263.5189	−2752.3562
	BQPSO	−1810.3356	−1498.0555	157.2211	−1272.7048	−1454.7974
	SOGA	−3248.4725	−2913.3245	218.1944	−2467.9053	−2952.6730

*F*_8_	BPSO	4.5456	7.6315	2.0230	15.5215	7.8838
	BQPSO	0.9446	1.9230	0.3842	2.5818	1.9273
	SOGA	0.0040	1.3614	1.1703	3.1276	1.8407

*F*_9_	BPSO	2.6925	12.6817	7.3535	31.3502	10.9785
	BQPSO	0.7294	1.7635	0.5748	2.9455	1.7687
	SOGA	0.0742	1.4639	1.4386	5.2594	0.9397

*F*_10_	BPSO	1.5798	3.6606	1.5582	7.7680	3.4519
	BQPSO	0.3552	0.8360	0.2063	1.1561	0.8923
	SOGA	0.0546	0.3702	0.1933	0.6738	0.4183

**Table 3:** Minimization results for SOGA and GA.

Function	Algorithm	The best	Mean	SD	The worst	Median
*F*_1_	SOGA	7.4510*e* − 05	1.6641*e* − 04	4.8945*e* − 04	0.0028	7.4510*e* − 05
	GA	0.0013	76.0248	166.2636	664.4937	2.5246
	SOGA^*∗*^	7.4510*e* − 05	0.0074	0.0189	0.0773	0.0007
	GA^*∗*^	0.0016	1.1302	2.3717	11.0953	0.2626

*F*_2_	SOGA	0.0024	0.0026	0.0005	0.0049	0.0024
	GA	0.0031	0.3220	0.4294	1.3587	0.1172
	SOGA^*∗*^	0.0031	0.0069	0.0076	0.0433	0.1059
	GA^*∗*^	0.0220	0.1423	0.1452	0.7703	2.5471

*F*_3_	SOGA	263.9170	1919.6076	1078.9650	3782.1623	2175.0068
	GA	823.0982	3805.95462	1587.4665	8753.2186	3602.7854
	SOGA^*∗*^	0.0238	27.5765	95.7945	434.9900	0.5557
	GA^*∗*^	482.1519	4922.3981	2417.1198	10470.9239	4705.2068

*F*_4_	SOGA	0	0.1000	0.4026	2	0
	GA	0	94.3667	457.2142	2509	1
	SOGA^*∗*^	0	0	0	0	0
	GA^*∗*^	0	3.6333	7.5177	36	2

*F*_5_	SOGA	0.0031	0.2228	0.6241	3.1281	0.0275
	GA	9.3783	27.0193	11.2953	53.1297	27.1523
	SOGA^*∗*^	0.0458	0.1908	0.0952	0.4913	0.1831
	GA^*∗*^	6.4913	20.0783	10.5937	49.6017	18.9795

*F*_6_	SOGA	−77.7357	−76.2789	0.6355	−75.1676	−76.2048
	GA	−77.9857	−75.2636	1.8587	−69.1535	−75.5698
	SOGA^*∗*^	−78.3316	−77.2289	0.6240	−75.8823	−77.1074
	GA^*∗*^	−77.5952	−74.8886	1.902	−70.5101	−75.457

*F*_7_	SOGA	−3248.4725	−2642.8652	256.1202	−2001.7012	−2952.6730
	GA	−3113.4015	−2088.3848	342.5752	−1355.6609	−2692.5141
	SOGA^*∗*^	−3351.7352	−3111.4602	154.6330	−2736.5711	−3114.6197
	GA^*∗*^	−3150.5510	−2733.6038	180.6351	−2424.3367	−2726.4449

*F*_8_	SOGA	0.0040	1.3614	1.1703	3.1276	1.8407
	GA	1.8409	3.3475	1.8626	10.2185	2.6024
	SOGA^*∗*^	0.0040	0.0223	0.0216	0.0911	0.0141
	GA^*∗*^	0.3384	2.7764	0.9262	4.3458	2.7676

*F*_9_	SOGA	0.0742	1.4639	1.4386	5.2594	0.9397
	GA	1.5468	10.2883	7.6703	34.3495	8.7194
	SOGA^*∗*^	0.0348	0.6195	0.5317	1.9344	0.4575
	GA^*∗*^	0.6679	6.0007	4.0074	13.6647	5.7359

*F*_10_	SOGA	0.0546	0.3702	0.1933	0.6738	0.4183
	GA	0.1600	0.9633	1.3811	7.9350	0.6882
	SOGA^*∗*^	0.2550	0.4715	0.1208	0.7548	0.4758
	GA^*∗*^	0.1805	0.7492	0.2431	1.2999	0.7741

^*∗*^The crossover and mutation operation act on substring.

**Table 4:** Comparison of SOGA with other algorithms and GA^*∗*^ with GA.

Function	Test	SOGA				GA^*∗*^
		BPSO	BQPSO	GA	GA^*∗*^	GA
*F*_1_	*p* value	<0.0001	<0.0001	0.0151	0.0115	0.0166
	*h*	1	1	1	1	1
*F*_2_	*p* value	<0.0001	<0.0001	0.0001	<0.0001	0.0340
	*h*	1	1	1	1	1
*F*_3_	*p* value	0.0724	<0.0001	<0.0001	<0.0001	0.4280
	*h*	−1	−1	1	1	0
*F*_4_	*p* value	<0.0001	<0.0001	0.2633	0.0119	0.2816
	*h*	1	1	0	1	0
*F*_5_	*p* value	<0.0001	<0.0001	<0.0001	<0.0001	0.0171
	*h*	1	1	1	1	1
*F*_6_	*p* value	<0.0001	<0.0001	0.0006	<0.0001	0.4430
	*h*	1	1	1	1	0
*F*_7_	*p* value	0.0081	<0.0001	0.0004	0.0080	0.2582
	*h*	1	1	1	1	0
*F*_8_	*p* value	<0.0001	0.0002	<0.0001	<0.0001	0.2063
	*h*	1	1	1	1	0
*F*_9_	*p* value	<0.0001	0.3884	<0.0001	<0.0001	0.0043
	*h*	1	0	1	1	1
*F*_10_	*p* value	<0.0001	<0.0001	0.0291	<0.0001	0.4065
	*h*	1	1	1	1	0

^*∗*^The crossover and mutation operation act on substring.

**Table 5:** Comparison of SOGA^*∗*^ with other algorithms.

Function	Test	SOGA^*∗*^				
		BPSO	BQPSO	GA	GA^*∗*^	SOGA
*F*_1_	*p* value	<0.0001	<0.0001	0.0151	0.0120	0.0409
	*h*	1	1	1	1	−1
*F*_2_	*p* value	<0.0001	<0.0001	0.0002	<0.0001	0.0308
	*h*	1	1	1	1	−1
*F*_3_	*p* value	<0.0001	0.8561	<0.0001	<0.0001	<0.0001
	*h*	1	0	1	1	1
*F*_4_	*p* value	<0.0001	<0.0001	0.2629	0.0104	0.1555
	*h*	1	1	0	1	0
*F*_5_	*p* value	<0.0001	<0.0001	<0.0001	<0.0001	0.2768
	*h*	1	1	1	1	0
*F*_6_	*p* value	<0.0001	<0.0001	0.0002	<0.0001	0.3923
	*h*	1	1	1	1	0
*F*_7_	*p* value	<0.0001	<0.0001	<0.0001	<0.0001	<0.0001
	*h*	1	1	1	1	1
*F*_8_	*p* value	<0.0001	<0.0001	<0.0001	<0.0001	<0.0001
	*h*	1	1	1	1	1
*F*_9_	*p* value	<0.0001	<0.0001	<0.0001	<0.0001	0.0044
	*h*	1	1	1	1	1
*F*_10_	*p* value	<0.0001	<0.0001	0.0435	<0.0001	0.1231
	*h*	1	1	1	1	0

^*∗*^The crossover and mutation operation act on substring.
